# Advances in Antifungal Drug Development: An Up-To-Date Mini Review

**DOI:** 10.3390/ph14121312

**Published:** 2021-12-16

**Authors:** Ghada Bouz, Martin Doležal

**Affiliations:** Faculty of Pharmacy in Hradec Králové, Charles University, 50005 Hradec Králové, Czech Republic

**Keywords:** antifungals, drug discovery, drug repurposing, drug targets, invasive aspergillosis treatment

## Abstract

The utility of clinically available antifungals is limited by their narrow spectrum of activity, high toxicity, and emerging resistance. Antifungal drug discovery has always been a challenging area, since fungi and their human host are eukaryotes, making it difficult to identify unique targets for antifungals. Novel antifungals in clinical development include first-in-class agents, new structures for an established target, and formulation modifications to marketed antifungals, in addition to repurposed agents. Membrane interacting peptides and aromatherapy are gaining increased attention in the field. Immunotherapy is another promising treatment option, with antifungal antibodies advancing into clinical trials. Novel targets for antifungal therapy are also being discovered, allowing the design of new promising agents that may overcome the resistance issue. In this mini review, we will summarize the current status of antifungal drug pipelines in clinical stages, and the most recent advancements in preclinical antifungal drug development, with special focus on their chemistry.

## 1. Introduction

For decades, fungal infections have been difficult health conditions to treat. This fact can be attributed to the narrow spectrum and high toxicity of clinically used antifungals, long duration of treatment and the high emergence of resistance towards available agents. The seriousness of fungal infections was brought back to light during the unfortunate COVID-19 pandemic, in the form of secondary life-threatening infections in the intensive care units [1]. *Candida*, *Cryptococcus*, and *Aspergillus* are the most common causative organisms of life-threatening human fungal infections [2]. *Candida auris* is a multi-drug resistant fungus [3]. *Lomentospora prolificans* has intrinsic resistance towards all clinically used antifungals. *Aspergillus fumigatus* is becoming more resistant to treatment, making it more difficult to treat aspergillosis, with mortality rate reaching 100% in some cases. Early detection and treatment of fungal meningitis and chronic pulmonary aspergillosis can save millions of lives around the world. Fungal infections have become a silent crisis, and prompt efforts are needed before it is too late. In this review, we will highlight current state-of-the-art developments in antifungal pipeline, both in clinical and preclinical stages, with special focus on their chemistry, in order to provide the reader with a comprehensive, up-to-date source that will influence future synthetic efforts. 

## 2. Clinically Used Antifungals 

**Figure 1:**
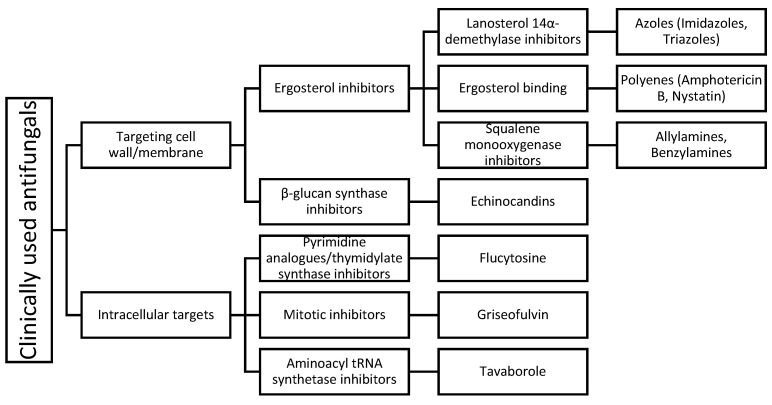
Clinically used antifungals grouped according to their mechanism of action with the most common agents in practice as examples.

The limited number of available antifungals with there narrow safety margin contributes to the increasing morbidity and mortality of invasive fungal infections. Clinically used antifungals can be classified according to their mechanism of action, as shown in Figure 1. 

**Figure 2:**
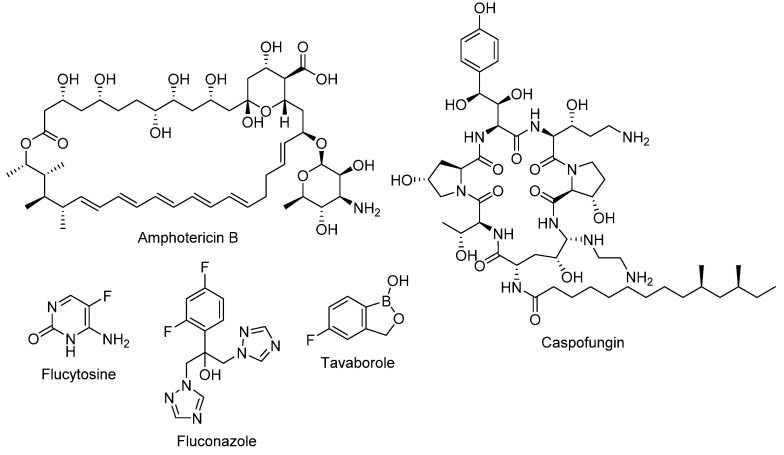
The chemical structures of amphotericin B (an example of a polyene); caspofungin (an example of an echinocandin); flucytosine; fluconazole (an example of an azole); and tavaborole.

Among all antifungals, only polyenes, flucytosine, azoles, and echinocandins are licensed for the treatment of invasive, life-threatening fungal infections (refer to Figure 2 for chemical structures) [4]. Amphotericin B is the clinically used polyene that is preserved as a second-line agent due to its high nephrotoxicity, which can lead, in some cases, to kidney failure. Several lipid-incorporated formulations of amphotericin B were developed as an attempt to reduce its nephrotoxicity; however, the high cost of such formulations limits their utility. Nystatin is a polyene macrolide antifungal that is used topically or orally to treat oropharyngeal candidiasis (for local effect, as it is not absorbed via the oral route) [5]. The major drawbacks of the pyrimidine antifungal flucytosine are the rapid development of resistance and high toxicity, both hepatological and hematological [6]. Due to their fungistatic mode of action, azoles are associated with a high rate of resistance, yet their wide safety margin contributes to their popularity. Azoles include compounds with either imidazole moiety, such as clotrimazole, or triazole moiety, such as fluconazole. Among the classical antifungals, the echinocandins were the last to be discovered in 1970s, taking them 30 years to progress from bench to bedside, to be marketed later in the year 2000 [7]. Although echinocandins have low rates of resistance, recently some fungi, especially non-albicans *candida*, started to develop resistance towards echinocandins by acquiring mutations [8]. The long stagnant phase in antifungal development ended with The Food and Drug Administration (FDA) approval of tavaborole in 2014 (refer to Figure 2 for chemical structure). Tavaborole is the first oxaborole and first tRNA synthetase-inhibitor antifungal to be approved for clinical use. It exerts its antifungal activity by inhibiting the cytosolic leucyl-transfer RNA synthetase (LeuRS), which plays a vital role in protein synthesis. It is licensed for topical use for the treatment of onychomycosis [9].

## 3. Novel Antifungals (In Clinical Settings)

**Figure 3:**
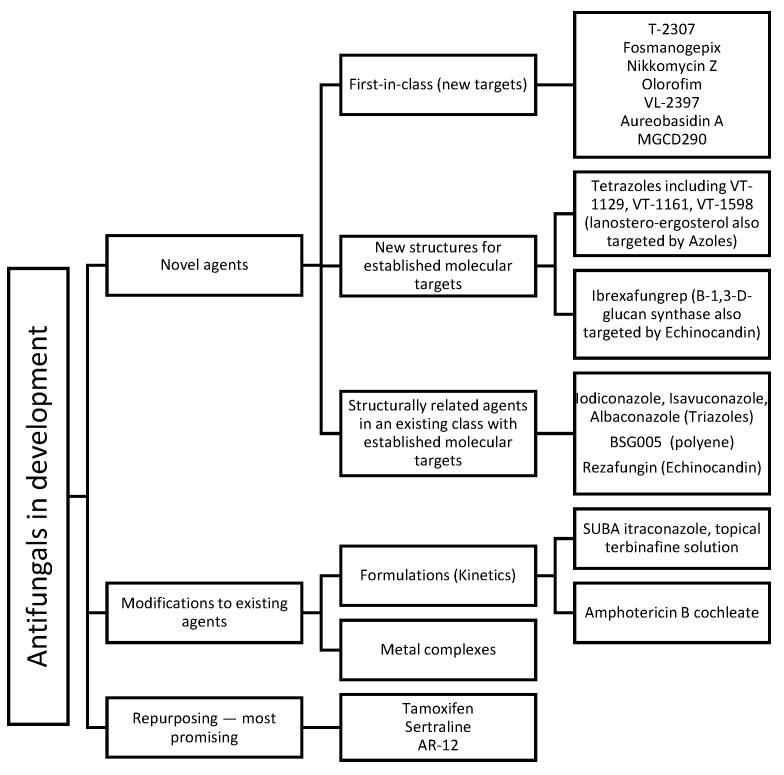
A summative figure showing the classification of new molecules as antifungals in clinical development.

The previously mentioned limitations of available antifungals urge the need for novel agents that can overcome these problems. Novel antifungals include first-in-class agents, new structures for a known target, new derivatives/analogues in an established class of drugs, and modification to the formulations of approved agents, in addition to repurposed agents (refer to Figure 3). Obtaining first-in-class designation significantly speeds up approval process and in-return market availability. Chemistry-wise, new antifungals with new structures include cyclic peptides (hexapeptides such as rezafungin and VL-2397 and depsipeptides such as aureobasidin A), triterpenoids (ibrexafungerp), tetrazoles (VT-1129, VT-1161, VT-1598), orotomides (olorofim), siderophores (VL-2397), and arylamidines (T-2307). Each agent will be discussed separately (ordered according to Figure 3) with a focus on their chemical aspects.

### 3.1. Aryldiamidines—T-2307

**Figure 4:**
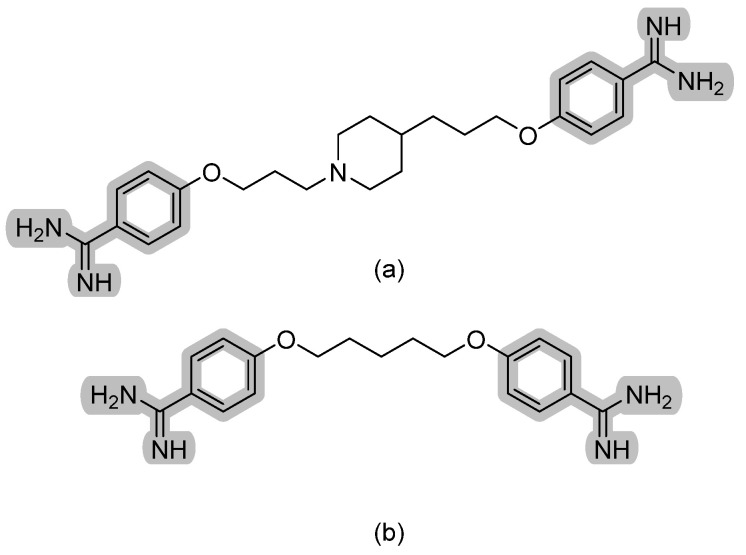
The chemical structures of (**a**) T-2307 and (**b**) pentamidine. The characteristic aryldiamidine moiety is highlighted in grey.

T-2307 is a first-in-class antifungal that exerts its fungicidal activity by inhibiting respiratory chain complexes and thus disrupting mitochondrial membrane potential. It is selectively transported into fungal cells through a polyamine transporter [10]. Structurally, it is an aromatic diamidine that is structurally related to the antiprotozoal agent pentamidine with a characteristic plane of symmetry. Pentamidine is used to treat pneumocystis, leishmaniasis, and trypanosomiasis by a similar mechanism (refer to Figure 4 for chemical structures). Pre-clinical data show T-2307 as very potent antifungal and a perhaps superior agent to azoles and polyenes in the treatment of invasive fungal infections [11]. Further information on T-2307 activity is summarized elsewhere [12]. 

### 3.2. Fosmanogepix and Manogepix

**Figure 5:**
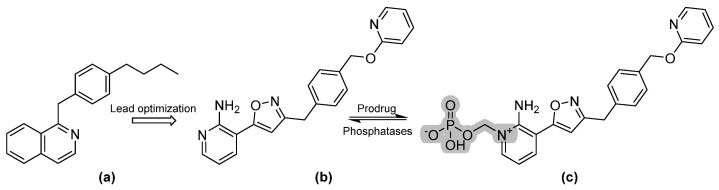
The chemical structures of (**a**) 1-[4-butylbenzyl]isoquinoline, (**b**) manogepix, and (**c**) fosmanogepix.

Fosmanogepix is the *N*-phosphonooxymethylene prodrug of manogepix (APX001A) that is hydrolyzed by systemic phosphatases (refer to Figure 5 for chemical structures). Manogepix was first identified during lead optimization studies to improve the potency of 1-[4-butylbenzyl]isoquinoline, a hit structure found to suppress the expression of surface glycosyl phosphatidylinositol (GPI)-mannoproteins, specifically the Gwt1 protein, in *Saccharomyces cerevisiae* and *Candida albicans*, and subsequently inhibit their growth [13,14]. Therefore, manogepix is a first-in-class antifungal that inhibits the fungal Gwt1 protein. Gwt1 is an enzyme that catalyzes inositol acylation, which is an early step in the glycosylphosphatidylinositol (GPI)-anchor biosynthesis pathway [15]. In 2019, fosmanogepix obtained orphan drug designation. Further information on fosmanogepix activity is reviewed elsewhere [16].

### 3.3. Nikkomycin Z

**Figure 6:**
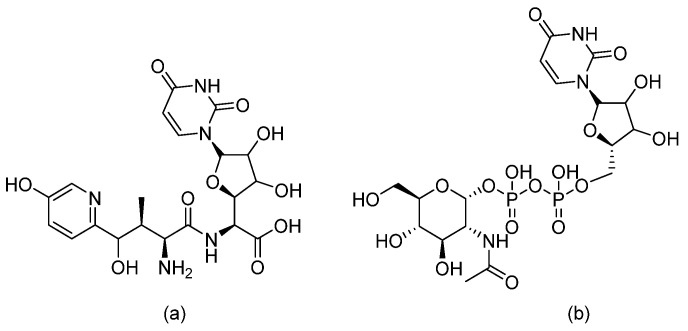
The chemical structures of (**a**) Nikkomycin Z and (**b**) Uridine Diphosphate (UDP)-*N*-acetyl glucosamine.

The discovery of nikkomycin Z goes back to the 70s. Structurally, nikkomycin Z resembles uridine diphosphate (UDP)-*N*-acetyl glucosamine, which is a precursor of chitin, and, thus, nikkomycin Z is a competitive inhibitor of chitin synthase (refer to Figure 6 for chemical structures) [17]. As a stand-alone drug, nikkomycin Z has poor in vitro fungicidal activity against Candida. However, taking into consideration that chitin is a major component of fungal cell walls, nikkomycin Z has synergistic activity with other antifungal cell-wall inhibitors, such as echinocandins [18]. Further information on nikkomycin activity is summarized elsewhere [19]. 

### 3.4. Orotomides—Olorofim

**Figure 7:**
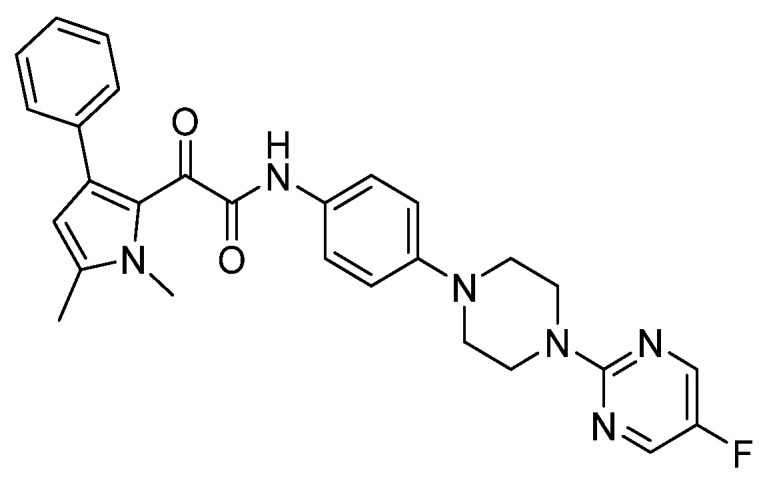
The chemical structures of olorofim.

The orotomides are a new class of antifungals that exert their fungicidal activity via a novel mechanism of targeting dihydroorotate dehydrogenase, which is a vital enzyme in fungal pyrimidine biosynthesis. Subsequently, nucleic acid and phospholipid synthesis is disrupted [20]. One agent belonging to this new class is olorofim (F901318). Olorofim is a potent antifungal agent that has time-dependent activity against drug resistant *Aspergillus* spp. and other uncommon molds with promising results in invasive and refractory fungal infections (refer to Figure 7 for chemical structure) [21]. Olorofim is 2000-fold more selective toward fungal dihydroorotate dehydrogenase than the human enzyme homologue [20]. Having a unique mechanism of action and potent antifungal activity against hard-to-treat fungi granted olorifim orphan drug designation (ODD) by the U.S. Food and Drug Administration (FDA) in March 2020 for the treatment of invasive aspergillosis and *Lomentospora/Scedosporium* infections and later in June 2020 for the treatment of coccidioidomycosis. Similarly, The European Medicines Agency Committee for Orphan Products granted orphan drug status to olorofim for the treatment of invasive aspergillosis and rare mold infections caused by *Scedosporium* spp. Further information on olorofim’s mechanism of action, pharmacokinetic profile, and clinical efficacy is summarized elsewhere [22].

### 3.5. Cyclic Peptides—VL-2397

**Figure 8:**
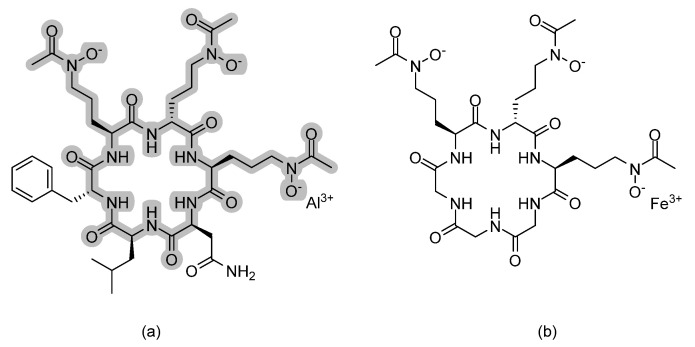
The chemical structures of (**a**) VL-2397 and (**b**) ferrichrome siderophore. To better visualize the structural resemblance, common fragments with ferrochrome siderophore are highlighted in grey.

VL-2397 (previously annotated as ASP2397) is a natural cyclic hexapeptide isolated from Acremonium persicinum cultures [23]. It was identified as a potential antifungal while screening various natural secondary metabolites in a silkworm infection model [23]. Structurally, VL-2397 resembles fungal ferrichrome siderophores (high-affinity, iron-chelating compounds secreted from microorganisms that serve as transporters to transport iron across cell membranes) and enters the fungal cells through specific transporters known as siderophore iron transporter 1 (Sit1) (refer to Figure 8 for chemical structures) [24]. Sit1 transporters are not present in mammalian cells, limiting possible toxicity to human cells. Once inside the cell, VL-2397 disrupts important intracellular processes through unknown mechanisms, and hence exerts its fungicidal activity [24].

### 3.6. Cyclic Peptides—Aureobasidin A

**Figure 9:**
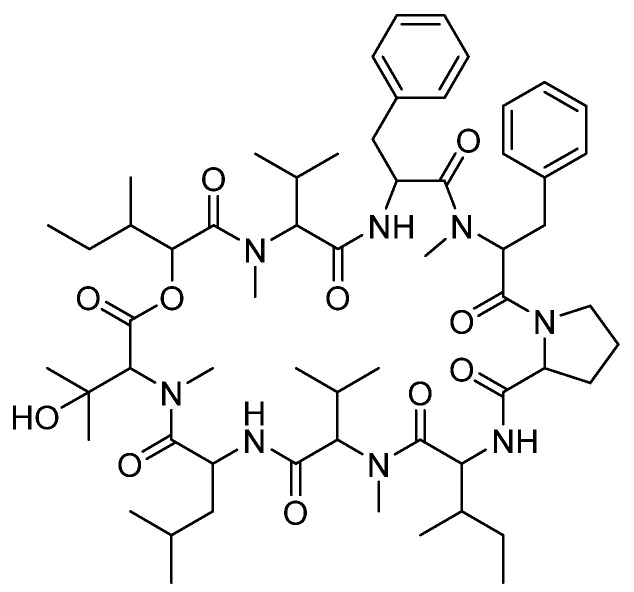
The chemical structure of Aureobasidin A.

Aureobasidin A is a natural cyclic depsipeptide isolated from the fungus *Aureobasidium pullulans* R106 that targets the essential inositol phosphorylceramide (sphingolipid) synthase in fungi (refer to Figure 9 for chemical structure) [25]. Interestingly, this broad-spectrum antifungal also exerts significant antiprotozoal activity against the proliferative tachyzoite form of Toxoplasma [26]. Structurally, Aureobasidin A consists of eight α-amino acid units and one hydroxy acid unit. Upon acid hydrolysis, Ikai and colleagues identified these units to be 2(*R*)-hydroxy-3(*R*)-methylpentanoic acid, beta-hydroxy-*N*-methyl-L-valine, *N*-methyl-L-valine, L-proline, allo-L-isoleucine, *N*-methyl-L-phenylalanine, L-leucine, and L-phenyl-alanine [25]. Several Aureobasidin A analogues have been prepared by Kurome and colleagues. They found that analogues with 4–6 carbon chain ester derivatives at the γ-carboxyl group of Glu6 or aIle6 exhibited the best antifungal activity [27]. 

### 3.7. MGCD290

MGCD290 (chemical structure is not presented in the literature) is an oral fungal Hos2 histone deacetylase (HDAC) inhibitor that also targets non-histone proteins, such as Hsp90, all of which play important roles in gene regulation [28]. Interfering with such fungal proteins suppresses fungal stress responses, which favors the use of MGCD290 as an adjuvant therapy to cell wall/membrane inhibitors [29]. Despite the in vitro synergic activity between MGCD290 and azoles/echinocandins, MGCD290 failed to show clinical importance in clinical settings, and hence its further development was suspended after a phase II clinical trial [28,30].

### 3.8. Tetrazoles

**Figure 10:**
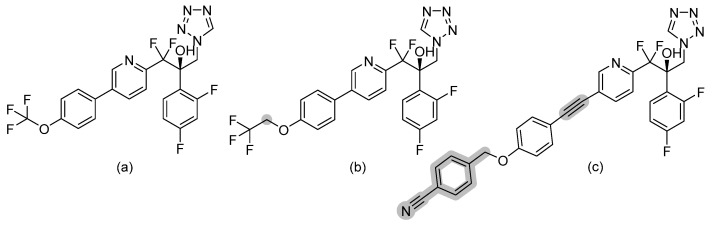
Chemical structures of (**a**) VT-1129, (**b**) VT-1161, and (**c**) VT-1598. Differences in chemical structures are highlighted in grey.

Tetrazoles are a new azole-like group bearing difluoromethyl-pyridines moiety, including the agents VT-1129 (quilseconazole), VT-1161 (oteseconazole), and VT-1598 (refer to Figure 10 for chemical structures). Tetrazoles are more selective to fungal CYP51 (lanosterol demethylase) rather than human enzymes, unlike triazoles, and hence have lower side effects and drug–drug interactions than triazoles [31]. VT-1161 and VT-1598 form H-bonds with the His377 of *Candida albicans* CYP51 and with the His374 of *Aspergillus fumigates* CYP51B [32]. The rationale behind the design of tetrazoles by W. J. Hoekstra et al. was to replace the metal binding group in triazoles (MBG, which is triazole) which has strong MBG/metal interaction with another MGB that has weaker MBG/metal interaction (tetrazole), as an attempt to improve selectivity. VT-1129 is a potent oral inhibitor for *Cryptococcus* species, including drug resistant strains with half-lives of six days [33]. VT-1161 is being investigated as a promising antifungal agent against both fluconazole-sensitive and fluconazole-resistant *Candida albicans* with clinical application in treating tinea pedis, onychomycosis, and vaginal candidiasis [34]. VT-1161 has half-life of 48 h [34]. VT-1598 was found to have anticandidal activity, especially against *Candida auris* [35].

### 3.9. Triterpenoids—Ibrexafungerp

**Figure 11:**
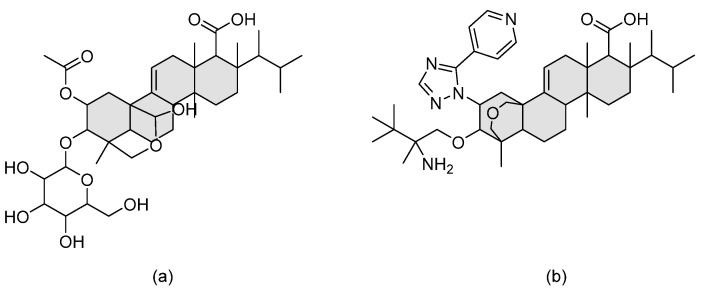
The chemical structures of (**a**) enfumafungin and (**b**) ibrexafungerp. The triterpenoid unit is shaded in grey.

During high-throughput screening of natural products produced by endophytic fungi, enfumafungin was identified as a promising antifungal hemiacetal triterpenoid glycoside. Structural modifications to improve pharmacokinetic properties and, most importantly, oral bioavailability led to the development of the semi-synthetic derivative ibrexafungerp (previously known as SCY-078 or MK-3118) (refer to Figure 11 for chemical structure). Ibrexafungerp exerts its antifungal activity, similarly to echinocandins, via inhibiting β-glucan synthase, yet it has a distinct chemical structure. It is fungicidal against *Candida* spp. and fungistatic against *Aspergillus* spp. [36]. Echinocandins are administered intravenously due to their poor per oral stability, which necessitates switching the patient to an oral, and perhaps less potent, antifungal upon discharge. Nevertheless, there is the emerging problem of resistance that urges the need for a safer agent that can be administered at higher doses. Thus, being an orally administered agent, ibrexafungerp is superior to known echinocandins when it comes to patients’ convenience. 

### 3.10. Triazoles

**Figure 12:**
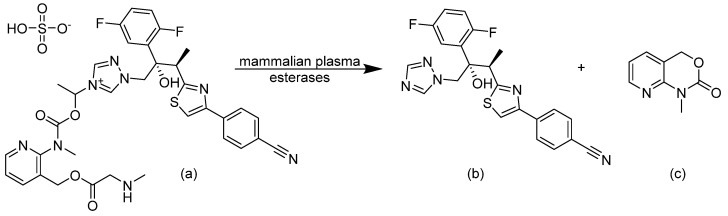
Hydrolysis reaction of the prodrug (**a**) isavuconazonium by mammalian plasma esterases to yield, (**b**) isavuconazole, and (**c**) by-product.

Since they are the most widely used antifungal class, there were several attempts to improve the efficacy of triazoles. Isavuconazonium sulfate is a water-soluble prodrug of isavuconazole, suitable for both oral and IV administration [37]. The [*N*-(3-acetoxypropyl)-*N*-methylamino]-carboxymethyl group is linked by an ester functionality to the triazole nitrogen of isavuconazole. The prodrug is cleaved by human plasma esterases releasing isavuconazole and low levels of cleavage by-product (refer to Figure 12) [38]. Isavuconazole has long half-life, broad spectrum of activity, and good efficacy [38]. It was FDA approved in 2015 for the treatment of aspergillosis and invasive mucormycosis. 

**Figure 13:**
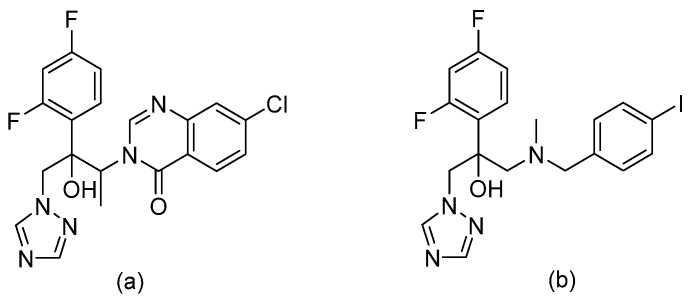
The chemical structures of (**a**) albaconazole and (**b**) iodiconazole.

Albaconazole is another 7-chloro triazole under development for the treatment of acute candida vulvovaginitis and onychomycosis (refer to Figure 13 for chemical structure). It has improved pharmacokinetic properties that resulted in excellent oral bioavailability [39]. 

Iodiconazole is a new topical triazole for the treatment of dermatophytosis in humans (refer to Figure 13 for chemical structure). It has a broad spectrum and potent activity against different fungal strains [40].

### 3.11. BSG005 

**Figure 14:**
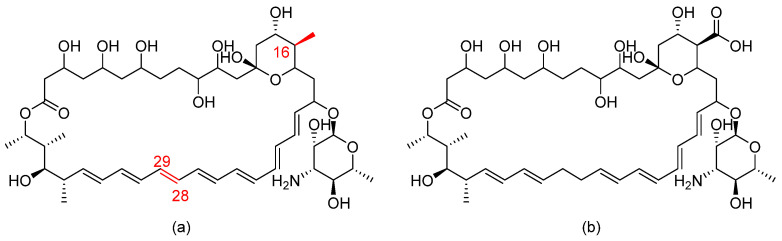
The chemical structures of (**a**) BSG005 and (**b**) nystatin. The structural modifications are shown in red.

BSG005 is a natural antifungal isolated from *Streptomyces noursei*. Structurally, BSG005 is an improved version of the polyene nystatin A (heptaene nystatin analogue) (refer to Figure 14 for chemical structures) [41]. It exerts fungicidal activity against a wide range of fungal strains, including azole- and echinocandin- resistant *Aspergillus* spp. and *Candida* spp., by a similar mechanism to nystatin. BSG005 does not cause nephrotoxicity, overcoming the main drawback of polyenes [41]. 

### 3.12. Cyclic Peptides—Rezafungin

**Figure 15:**
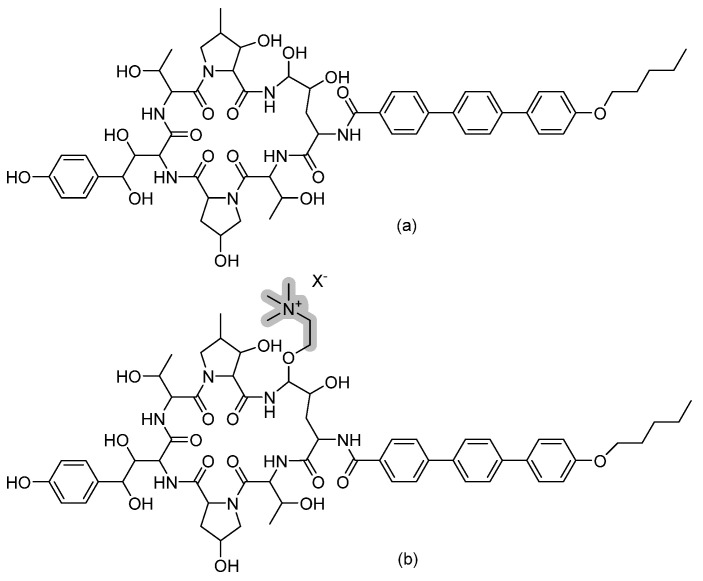
The chemical structures of (**a**) anidulafungin and (**b**) rezafungin. The chemical modification in the structure of rezafungin over anidulafungin is highlighted in grey.

Rezafungin (previously known as CD101) is a novel echinocandin, another cyclic hexapeptide derivative. It is an analogue of the echinocandin anidulafungin obtained by replacing the hemiaminal region at the C5 ornithine position of anidulafungin with choline ether (refer to Figure 15). This chemical modification led to an increased stability towards host degradation and subsequently prolonged the half-life, allowing once-weekly dosing [42]. When incubated in human plasma for 44 h at 37 °C, rezafungin showed a stability of 79–94%, while anidulafungin had 7–15% stability [43]. The prolonged pharmacokinetic property of rezafungin prolonged tissue exposure and hence permitted the use of rezafungin in prophylaxis regimens instead of the regular azoles [44]. This additional choline ether prevented the formation of toxic intermediates, favoring rezafungin safety and allowing its administration at higher doses to prevent resistance [43]. 

### 3.13. SUBA Itraconazole 

Super-bioavailability-itraconazole (SUBA itraconazole) overcame the low bioavailability issue of itraconazole by dispersing itraconazole in a pH-dependent polymer matrix as capsules. This change to the formulation increased the bioavailability of itraconazole by 173% [45]. SUBA itraconazole was granted FDA approval in 2018 for the treatment of aspergillosis, histoplasmosis, and blastomycosis in patients with contraindications to the use of amphotericin B (intolerant or refractory). 

### 3.14. Topical Terbinafine Solution 

Oral terbinafine is a gold-standard treatment for onychomycosis; however the treatment is lengthy, as it takes terbinafine a long time to concentrate in the nail plate and bed, increasing the potential for the development of systemic side effects and resistance. One solution was to prepare terbinafine as a solution for topical use (MOB-01) [46]. Pharmacokinetic studies showed that, when compared with oral terbinafine, topical terbinafine achieves higher concentrations in the nail plate (≈10,003 times more) and nail bed (≈403 times more) with limited or no systemic absorption. Similar examples include two new ophthalmic solutions for the treatment of candida infections; one containing hexamidine diisethionate 0.05% (keratosept) [47], and the other containing povidone-iodine 0.6% (IODIM^®^) [48].

### 3.15. Amphotericin B Cochleate 

Amphotericin B cochleate is a polyene formulation of amphotericin B that is stable against gastric degradation and hence is suitable for oral administration [49]. Cochleates are made up of phosphatidylserine with phospholipid-calcium precipitates in a multilayer system that has spiral configuration. When the cochleates reach the blood stream, the spiral structure opens once the calcium concentration drop in the cochleate, releasing the encapsulated amphotericin B [49]. The possibility to administer amphotericin B via the oral route overcame infusion-related complications and offered a practical, patient-convenient, broad-spectrum antifungal treatment. 

### 3.16. Metal Complexes and Chelates 

**Figure 16:**
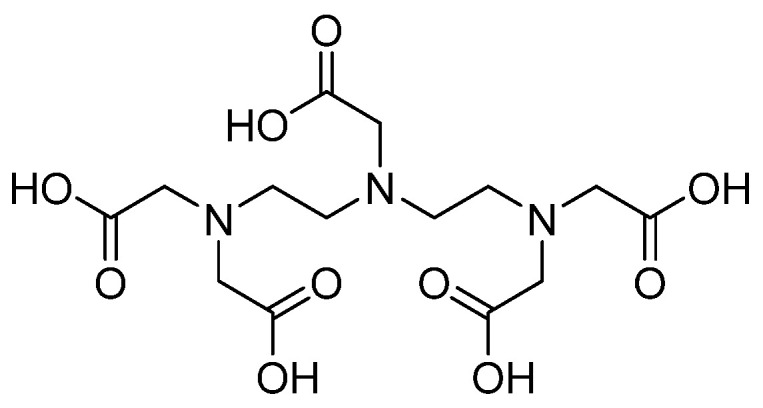
The chemical structure of the broad-spectrum chelator diethylenetriamine pentaacetate (DTPA).

Metal complexes may be a novel promising class of antifungals due to improved properties, especially those related to stereochemistry, redox potential, and lipophilicity [50]. Such an approach may provide an easy solution to overcome the issue of fungal resistance. Successful examples of established antifungals complexed with metal ions include organoruthenium complexes conjugated with three different azoles (namely clotrimazole, tioconazole, and miconazole) reported by Kljun et al. [51]. The resulting mono-, bis-, and tris–azoles complexes had millimolar inhibitory concentrations against *Culvularia lunata*. Another example is reported by Stevanovic et al., who showed that a fluconazole zinc (II) complex had significantly better antifungal activity against *Candida krusei* and *Candida parapsilosis* than fluconazole [52]. Other new metal coordinates, not complexed with known antifungals, are at preclinical stages [53,54]. On the other hand, Polvi and colleagues screened a library of pharmacologically active compounds that do not themselves possess antifungal activity as an attempt to identify compounds that can potentiate the efficacy of caspofungin against echinocandin-resistant *Candida albicans* strains. They identified the broad-spectrum chelator diethylenetriamine pentaacetate (DTPA) as a promising compound that synergizes with capsofungin (refer to Figure 16 for chemical structure). They justified this potentiating activity of DTPA by magnesium chelation [55]. The potential of metal-based compounds as antifungals is thoroughly reviewed elsewhere [56].

The current status for each of the antifungals discussed in this review, including the name of companies and clinical trial registration numbers, are summarized in Table 1 below.

## 4. New Compounds as Potential Antifungals (In Preclinical Stages) 

Several research groups, both in academia and the industry, are focused on developing new, potentially active antifungals. We will briefly mention some of the most recent (published in 2021), successful synthetic efforts from academia (refer to Table 2 for general structures). 

Ravu and colleagues recently reported the synthesis and in vitro antifungal evaluation of a series of phloeodictine analogues [59]. Phloeodictines are marine-derived alkaloids, and the phloeodictine-based 6-hydroxy-2,3,4,6-tetrahydropyrrolo[1,2-*a*]pyrimidinium moiety with an *n*-tetradecyl side chain at C-6 has shown antifungal activity and serves as a template for further derivatization. They identified three promising analogues (compounds 24, 36, and 48 in the original paper) with potent activity (MIC ≈ 1 µM) against *Candida neoformans* and low toxicity against mammalian Vero cells (IC_50_ > 40 μM) [59].

Choi et al. tested their in-house library where they identified a 2-amino-*N*-(2-(3,4-dichloro-[1,1-biphenyl]-4-yl)ethyl)-pentanamide hydrochloride (compound 22h in the original paper) as a promising hit with potent, fast fungicidal activity against *Candida neoformans* (MIC ≈ 2.5 µM) and *Candida albicans* (MIC ≈ 5 µM) [60]. The latter compound also showed synergistic in vitro activity with clinically available antifungals and potent in vivo efficacy in a subcutaneous infection mouse model and an ex vivo human nail infection model. The authors claim that their hit compound exerts its antifungal activity by interfering with fungal cell wall integrity.

On the other hand, Kato et al. prepared thiazoyl guanidine derivatives that inhibit fungal ergosterol biosynthesis [61]. Their hit compound *N*-(2′-(4-(methylsulfonyl)phenyl)-[4,4′-bithiazol]-2-yl)-tetrahydropyrimidin-2(1H)-imine (compound 6h in original paper) is structurally related to the antifungal abafungin and showed potent in vitro antifungal activity against *Aspergillus fumigatus* (MIC = 4.7 μM) with a favorable pharmacokinetic profile. In addition, the latter compound exhibited antifungal activity comparable to voriconazole in a murine model of *Aspergillus fumigatus* infection.

Furthermore, Li and colleagues prepared a series of carboline fungal histone deacetylase (HDAC) inhibitors in an attempt to develop a promising compound for the combinational treatment of azole-resistant candidiasis [62]. Among all synthesized compounds, 2-(4-(3-(8-chloro-1,2,3,4-tetrahydro-9H-pyrido[3,4-b]indol-9-yl)propoxy)phenyl)-*N*-hydroxyacetamide (compound D12 in original paper) showed excellent in vitro and in vivo synergistic antifungal activity with fluconazole to treat azole-resistant candidiasis.

Fungal fatty acid (FA) synthase and desaturase (responsible for introducing double bonds to yield unsaturated FAs) are essential enzymes for the growth and virulence of fungal pathogens [63]. They are structurally distinct from their mammalian homologues, making them suitable targets for antifungal development. DeJarnette et al. performed whole-cell screening using *Candida albicans* with varying levels of FA synthase or desaturase [64]. They identified four acyl hydrazides as the most promising candidates (compounds 2, 40, 41, and 48 in the original paper) with broad-spectrum activity against *Candida albicans*, *Candida auris*, and mucormycetes, including activity against azole-resistant *Candida* and low in vitro cytotoxicity in HepG2 liver cancer cell line.

Lastly, Lowes and colleagues designed and synthesized dual inhibitors targeting fungal acetohydroxy-acid synthase and NLRP3 inflammasome [65]. Their design rationale was based on the structural similarities between the two types of inhibitors. Fungal acetohydroxy-acid synthase is the common enzyme in the branched-chain amino acid-synthesis pathway [66], while NLRP3 inflammasome is a mammalian cytosolic receptor that mediates innate inflammatory responses to offending fungi by releasing mature interlukein-1 beta (IL-1β) upon activation [67]. The authors established the essential molecular scaffold required for dual activity for the first time, which shall serve as a template for future synthetic efforts. Among their prepared inhibitors, they identified 2-((1-(4-fluorophenyl)-3-oxo-3-phenylpropyl)thio)benzoate (compound 10 in original paper) as the most promising dual inhibitor, since it significantly decreased IL-1β release (IC_50_ = 2.3 ± 0.8 μM) without affecting mammalian cell viability (viability = 101.5 ± 1.4%) and exerted potent in vitro antifungal activity against *Candida albicans* (MIC = 6.4 ± 2.6 μM).

Aside from the mentioned small molecules with intracellular targets, membrane-interacting antifungal antimicrobial peptides (AMPs) are another example of a promising class of antifungals. AMPs generally consist of 12 to 54 amino acids with a net positive charge at physiological pH [68]. They are amphipathic in nature, which enhances their interaction with target membranes [69]. Since they target fungal plasma membranes or cell walls, they have the advantage of avoiding intracellular resistance mechanisms. AMPs are selective, with multiple modes of action (for example interaction with membrane phospholipids, sphingolipids, or proteins), and low toxicity to mammalian cells. Detailed information on antifungal AMPs has recently been reviewed elsewhere [70].

In addition, several studies were conducted to explore the role of essential oils in treating fungal infections. In their recent work, Donadu et al. investigated the antifungal activity of the essential oil of the Colombian rue, *Ruta graveolens* (REO) [71]. They found that REO exerts fungicidal activity against *Candida tropicalis* and fungistatic activity against *Candida albicans* by disrupting cellular membrane integrity. REO also showed synergistic activity with amphotericin B [71]. Furthermore, an oil macerate of *Helichrysum microphyllum Cambess*. subsp. *tyrrhenicum Bacch*., *Brullo* & *Giusso* showed potent inhibiting activity on candida growth, making it a promising agent to be used topically in the treatment of candidiasis [72]. Another example is the essential oil of *Austroeupatorium inulaefolium*, which showed strong species-dependent antifungal activity against *Penicillium brevicompactum* and *Fusarium oxysporum* [73].

## 5. Repurposing

**Figure 17:**
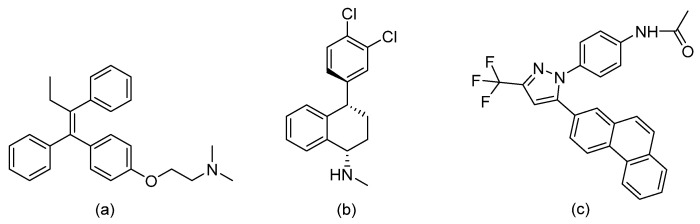
The chemical structures of (**a**) tamoxifen, (**b**) sertraline, and (**c**) AR-12.

Repurposing established drugs with possible antifungal activity and pushing them into the antifungal development pipeline saves time and resources, especially given that the pharmacokinetic and pharmacodynamic profiles are already known for such agents. The most promising candidates to be used in antifungal regimens are the selective estrogen receptor modulator tamoxifen (used for breast cancer) and the serotonin reuptake inhibitor sertraline (used for depression) (refer to Figure 17 for chemical structures). Tamoxifen showed anticryptococcal activity and may be a promising synergistic agent with fluconazole [74]. The most advanced repurposing attempt is for sertraline. An ongoing phase III trial is investigating the role of sertraline as an adjuvant therapy to the standard treatment for cryptococcal meningitis (NCT01802385). Furthermore, AR-12 is a celecoxib derivative that was assessed for safety in a phase I oncology clinical trial (refer to Figure 16 for chemical structure). However, it showed consistent antifungal activity against certain yeasts and molds, and thus it was repurposed as a promising adjuvant therapy to fluconazole in the treatment of invasive fungal infections [75].

## 6. Immunotherapy

Immunotherapy is a new promising strategy to modulate the host immune system and strengthen the innate and adaptive immune response to fight fungal infections. Immunotherapy approaches in treating fungal infections include the administration of recombinant growth factors and cytokines, granulocyte and granulocyte-macrophage colony-stimulating factors, and antibodies. Immunocompromised patients can also benefit from cell therapy, during which innate and adaptive immune cells are introduced to enhance the immune response against the offending fungi. In this review we will focus on antifungal antibodies, which are also known as antifungal passive immunotherapy. Other immunotherapy approaches are reviewed elsewhere [76].

Currently, there only two antifungal antibodies in clinical development. MAb 2G8 is a monoclonal antibody that targets laminarin (consisting mainly of β glucans). It binds to the walls of *Candida albicans* and *Cryptococcus neoformans*, inhibiting their growth and capsule formation [77]. Mambro and colleagues reported the development of a new humanized monoclonal antibody derived from MAb 2G8 that specifically targets β-1,3 glucans of pathogenic fungi, such as *Candida* spp. [78]. The new derivative showed potent in vitro antifungal activity against *Candida auris*.

Efungumab (also known as mycograb) is a single-chain variable-fragment antibody that targets heat shock protein 90 (HSP90). Efungumab was tested in combination with amphotericin B in clinical settings, where it showed a reduction in the mortality and an improvement in the survival of patients infected with *Candida*. It must be noted that efungumab was also investigated in clinical trials as an adjuvant to docetaxel in patients with breast cancer (NCT00217815).

One step beyond antifungal antibodies is radio-immunotherapy, where the antifungal antibody is linked to radioisotopes in order to specifically release fungicidal radiation in fungal cells [79]. This approach showed promising results in treating drug-resistant *Cryptococcus neoformans* infections [79].

## 7. New Promising Targets for Antifungal Development

Their classification as eukaryotes makes fungal infections a more challenging condition to treat in the drug discovery pipeline. It is important to discover unique targets that are present in fungi and not in humans in order to improve the selectivity and subsequently the safety profile of such agents. One interesting approach to discover new antifungals is what Novartis did by screening their chemical dark matter database for potential antifungals. Chemical dark matter libraries include molecules that have no bioactivity in human targets and thus are of increased value in screening for active molecules against other eukaryotes. During their screening campaign, Novartis have identified a novel antifungal agent and a novel fungal target pathway, which is hemebiosynthesis. The identification of molecular targets opened the window for medicinal chemists to design inhibitors following a target-based drug design approach.

**Figure 18:**
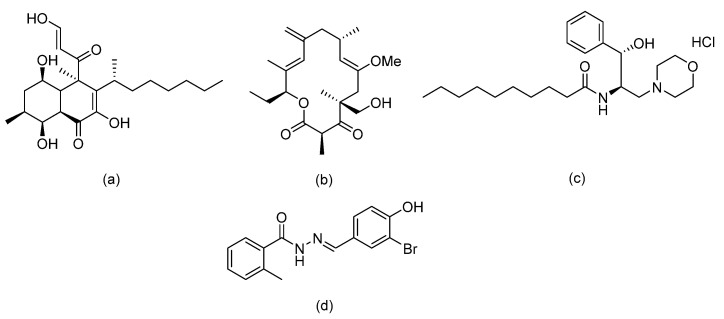
The chemical structures of (**a**) australifungin, (**b**) galbonolide A, (**c**) D-threo-PDMP, and (**d**) BHBM.

Another example of a new, promising fungal pathway for antifungal drug development is a sphingolipid synthesis pathway. Sphingolipid synthesis is highly preserved among eukaryotes; however, a number of vital structures are unique to fungi, making them promising treatment targets. In fungi, sphingolipids such as inositolphosphoryl ceramide (IPC) and glucosylceramide (GlcCer) play an important role in fungal pathogenicity and fungal growth [80]. Structures that inhibit the enzymes responsible for the synthesis of the latter two sphingolipids were found to disrupt the virulence of *Candida albicans*, *Cryptococcus neoformans*, and *Aspergillus* spp. [81,82]. Promising pre-clinical molecules targeting sphingolipid biosynthesis include sphingosine *N*-acyltransferase (e.g., australifungin) [83], IPC inhibitors (e.g., the antifungal non-glycosidic macrolide galbonolide A (also known as rustmicin)) [84], GlcCer synthase inhibitors (e.g., D-threo-PDMP) [81], and GlcCer inhibitors (e.g., BHBM) [85], among others (refer to Figure 18 for chemical structures). For such agents, human toxicity is of great concern, except for IPC inhibitors, since mammals do not express IPC [80].

In addition, Krystufek and colleagues described in their latest work the great potential of aspartic proteases, especially Major aspartyl peptidase 1 (May1) secreted from *Cryptococcus neoformans*, as a potential target for antifungal drug development [86]. Inhibitors of such targets shall be of great benefit, especially in treating cryptococcosis caused by *Cryptococcus neoformans* or *Cryptococcus gattii* in immunocompromised patients with HIV, since such inhibitors are also antiretrovirals. In addition to identifying a new promising target, the authors identified promising inhibitors derived from an *N*-terminally carboxybenzylated phenylstatine scaffold (refer to Figure 18e for general structure). Other fungal targets, such as the Ras pathway, trehalose pathway, metabolic glyoxylate cycle, high-osmolarity glycerol pathway, etc., are also under investigation.

## 8. Conclusions and Remarks

When comparing older antifungals with the newer ones mentioned in this review, one can sense the influence of the powerful recent advances in structural biology and medicinal chemistry on phenotypic target discovery, drug discovery, and target-based drug design. It must be noted that in order to truly evaluate the efficacy of new antifungals in treating life-threatening infections, infection-specific biomarkers should be relied on rather than death as an endpoint, since such targeted patients have multiple health comorbidities. By doing so, we ensure that no potential hit is lost as a false negative. One important observation from the examples of antifungals in development mentioned earlier in text is the presence of halogen atoms, especially fluorine and chlorine, in their chemical structures. This suggests the significant importance of halogen bonding for the interactions with their corresponding targets.

## Figures and Tables

**Table 1:** The spectrum of activity, route of administration, production company, and current status of antifungals in clinical development. Clinical registration numbers were obtained from www.clinicaltrials.gov (accessed on 26 November 2021).

Agent	Spectrum of Activity	Route of Administration	Company/Sponsor	Current Status	Clinical Trial Registration Number
T-2307	Broad spectrum	IV	Toyama Chemical Ltd.	Phase I completed (as stated in [57], but no actual data available in the literature)	Not available
Fosmanogepix	*Candida* spp. (*except C. krusei*) and *Aspergillus* spp.	PO/IV	Amplyx Pharmaceuticals	Phase II recruiting	NCT04240886
Nikkomycin Z	*Candida* spp. and *Aspergillus* spp.	PO	University of Arizona	Phase I completed; however lack of funding and volunteers caused the termination of phase II studies	NCT00834184
Olorofim	*Aspergillus* spp. and uncommon molds	PO/IV	F2G Ltd.	Phase II Phase III planned (not yet recruiting)	NCT03583164 NCT05101187
VL-2397	*Aspergillus* spp. and some *Candida* spp.	IV	Vical Biotechnology	No current development plans (phase II trial terminated early, because of a business decision)	NCT03327727
Aureobasidin A	Board spectrum and proliferative tachyzoite form of toxoplasma (antiprotozoal)	PO/IV	Takara Bio Group	Preclinical	Not available
MGCD290	*Candida* spp. and *Aspergillus* spp.	PO	MethylGene, Inc.	Further development suspended after phase II clinical trial	NCT01497223
VT-1129	*Candida* spp. and *Cryptococus* spp.	PO	Viamet Pharmaceuticals Inc.	Preclinical	Not available
VT-1161	*Candida* spp., *coccidioides* spp., and *Rhizopus* spp.	PO	Mycovia Pharmaceuticals	Phase III completed	NCT03561701
VT-1598	*Candida* spp., *Aspergillus* spp., and *Cryptococus* spp.	PO	Mycovia Pharmaceuticals Clinical trial sponsored by National Institute of Allergy and Infectious Diseases (NIAID)	Phase I	NCT04208321
Ibrexafungerp	*Candida* spp. and *Aspergillus* spp.	PO/IV	Scynexis, Inc.	Phase III	NCT03059992
Isavuconazole	Broad spectrum	PO (Isavuconazonium sulfate PO/IV)	Basilea and Astellas Clinical trials sponsored by Memorial Sloan Kettering Cancer Center and M.D. Anderson Cancer Center, respectively.	Phase II trials completed FDA approved in 2015 for the treatment of aspergillosis and invasive mucormycosis	NCT03149055 NCT03019939
Albaconazole	Broad spectrum	PO	Palau Pharma Clinical trial sponsored by GSK	Phase II completed	NCT00730405
Iodiconazole	Broad spectrum	Topical	Second Military Medical University and Anhui Jiren Pharmaceutical	Phase III (as stated in [58], but no actual data available in the literature)	Not available
BSG005	Broad spectrum	IV	Biosergen AS	Phase I	NCT04921254
Rezafungin	*Candida* spp., *Aspergillus* spp., and *Pneumocystis jirovecii*	IV	Cidara Therapeutics, Inc.	Phase III	NCT03667690 NCT04368559
SUBA-itraconazole	Broad spectrum	PO	Mayne Pharma Ltd.	Phase II FDA approved in 2018 for the treatment of aspergillosis, histoplasmosis, and blastomycosis in patients contraindicated to amphotericin B	NCT03572049
Topical terbinafine solution	Onychomycosis	Topical 10% solution	Moberg Pharma	Phase III	NCT02859519
Amphotericin B cochleate	Broad spectrum	PO	Matinas BioPharma	Phase II	NCT02629419

**Table 2:** The general structures of some of the most recent (published in 2021), successful synthetic efforts from academia.

Structural Type	General Structure *	Target	Ref.
Phloeodictine analogues	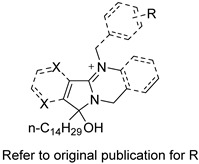	Fungal ergosterol biosynthesis	[59]
Biphenylethylaminoacetamides	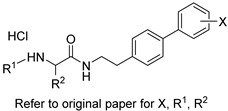	Fungal cell wall integrity	[60]
Thiazoyl guanidine derivatives	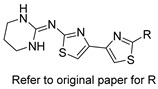	Fungal ergosterol biosynthesis	[61]
Carboline HDAC inhibitors	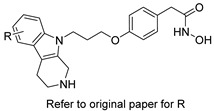	Fungal histone deacetylase	[62]
Acyl hydrazides	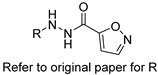	Fungal fatty acid biosynthesis	[64]
Thiobenzoate scaffolds	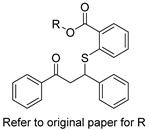	Dual target: fungal acetohydroxyacid synthase and NLRP3 inflammasome	[65]

* Adapted with permission from refs. [59,60,61,62,64,65]. Copyright 2021 American Chemical Society.

## Data Availability

Not applicable.
